# Supplementary material for: Hadza Prevotella Require Diet-derived Microbiota Accessible Carbohydrates to Persist in Mice
Source: bioRxiv. 2023 Mar 9:2023.03.08.531063. Preprint. [Version 1] doi: 10.1101/2023.03.08.531063 (PMC10028851; doi:10.1101/2023.03.08.531063)
Supplement: 1 [file NIHPP2023.03.08.531063V1-supplement-1.pdf]

bioRxiv preprint doi: https://doi.org/10.1101/2023.03.08.531063; this version posted March 9, 2023. The copyright holder for this preprint (which was not certified by peer review) is the author/funder, who has granted bioRxiv a license to display the preprint in perpetuity. It is made available under aCC-BY 4.0 International license.

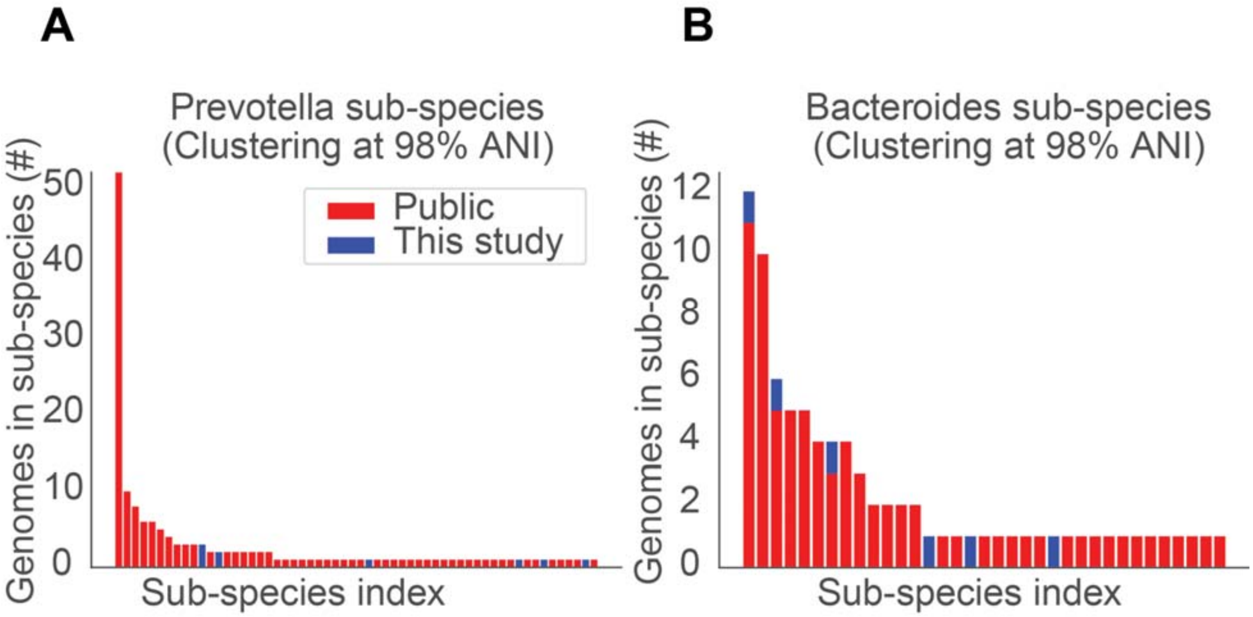

Figure S1. Clustering subspecies of *Bacteroides* and *Prevotella*. Number of representative genomes from *Prevotella* **(A)** and *Bacteroides* **(B)** subspecies used in the genome comparisons in **Fig. 1**. Sub-species index: genomes were clustered at 98% average nucleotide identity (ANI). Each bar represents one of these subspecies bins. Blue indicates genomes isolated for this study.

bioRxiv preprint doi: https://doi.org/10.1101/2023.03.08.531063; this version posted March 9, 2023. The copyright holder for this preprint (which was not certified by peer review) is the author/funder, who has granted bioRxiv a license to display the preprint in perpetuity. It is made available under aCC-BY 4.0 International license.

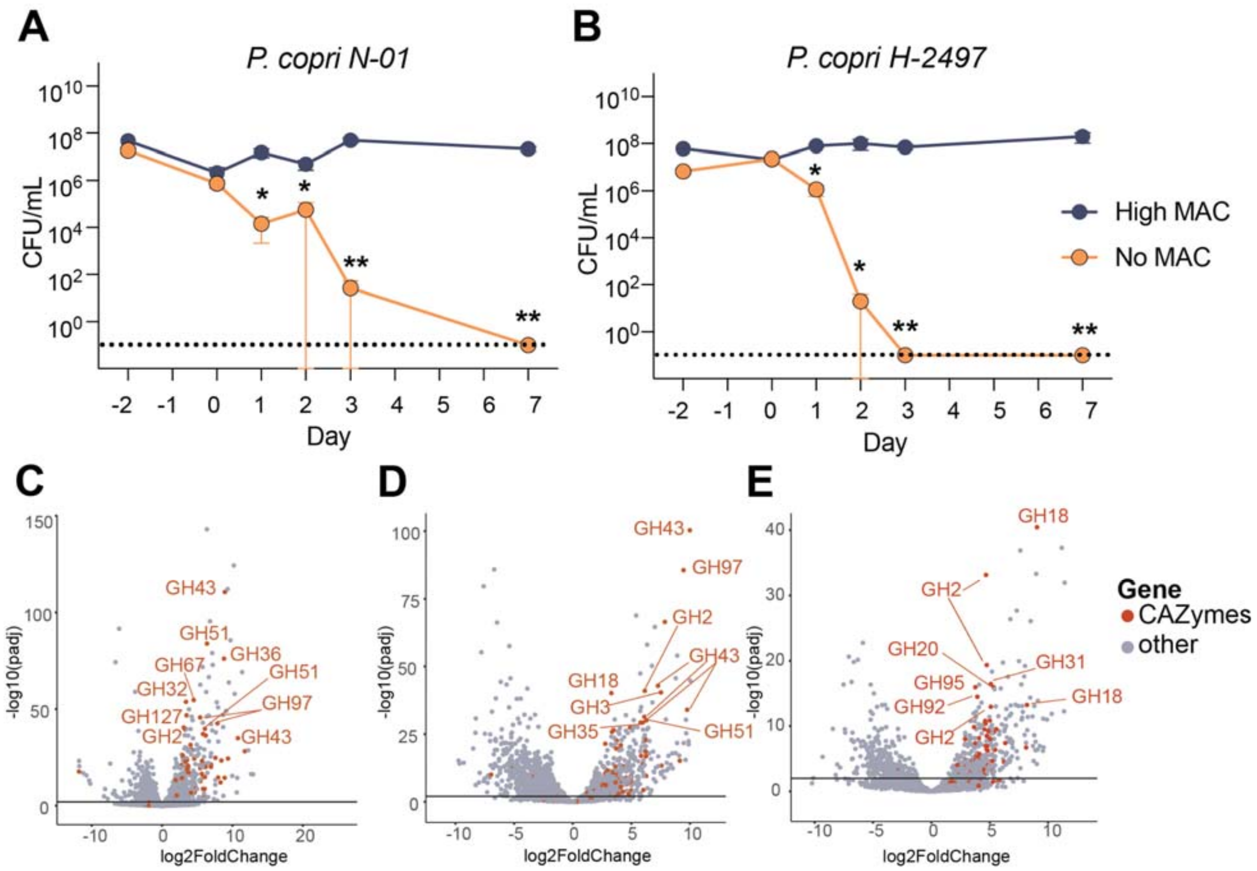

Figure S2. Diet-driven changes in *Bacteroides* and *Prevotella* colonization and gene expression. (A, B) Fecal density of *Pc N-01* **(A)**, and Hadza *Pc H-2497* **(B)**, in monocolonized mice fed different diets (Multiple Mann-Whitney tests, *: $p \leq 0.05$, **: $p \leq 0.01$). Dotted line indicates 0 CFU. Diet change to no MAC occurred on day 0. (**C, D)** Differential expression of *Pc H-2477* **(C)** or *Bt H-2622* **(D)** genes on High MAC *in vivo* vs. PYG *in vitro*. Black line at y=2. CAZyme genes marked in red. **(E)** Differential expression of *Bt H-2622* genes on No MAC *in vivo* vs. PYG *in vitro*. Black line at y=2. CAZyme genes marked in red.

bioRxiv preprint doi: https://doi.org/10.1101/2023.03.08.531063; this version posted March 9, 2023. The copyright holder for this preprint (which was not certified by peer review) is the author/funder, who has granted bioRxiv a license to display the preprint in perpetuity. It is made available under aCC-BY 4.0 International license.

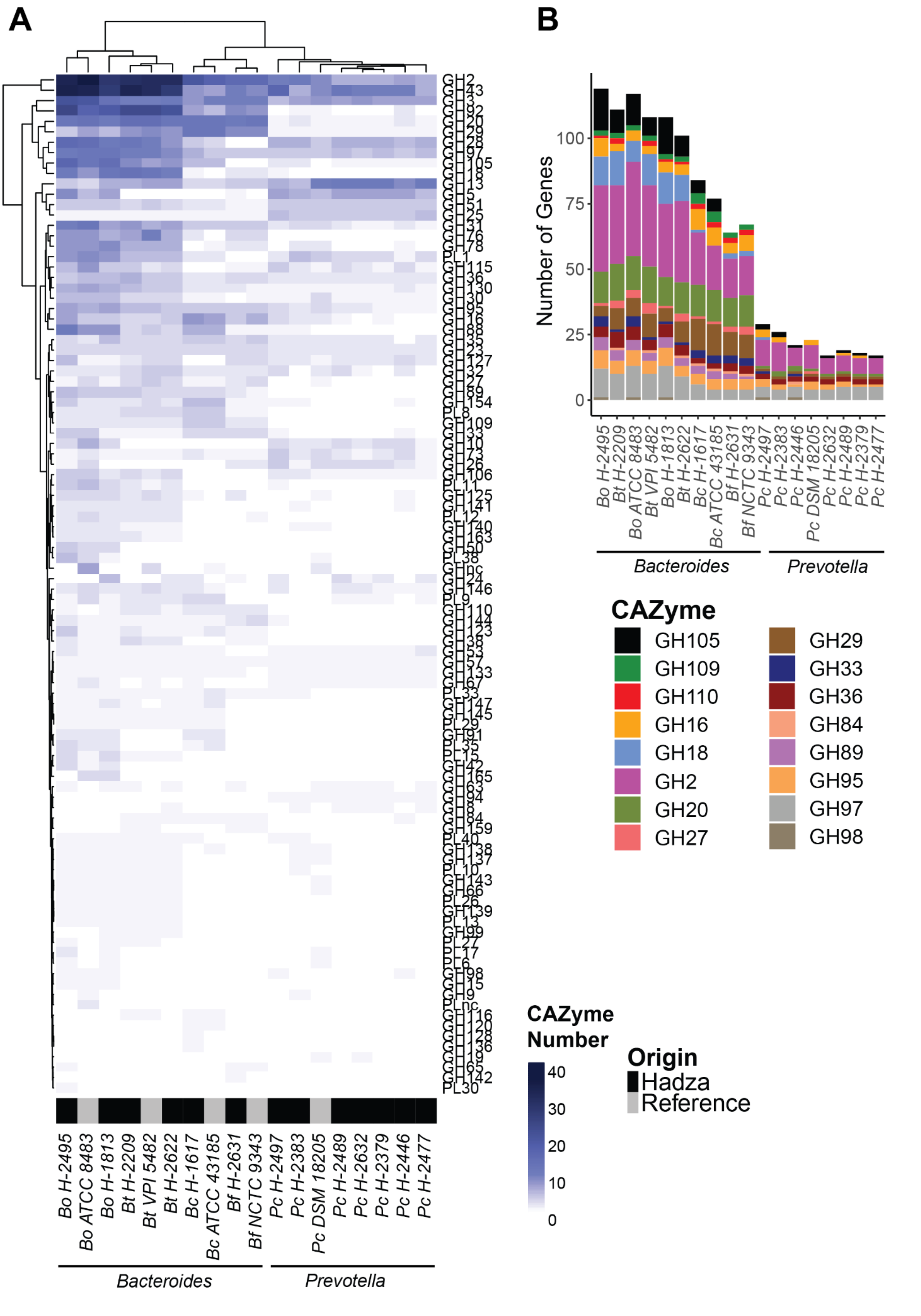

bioRxiv preprint doi: https://doi.org/10.1101/2023.03.08.531063; this version posted March 9, 2023. The copyright holder for this preprint (which was not certified by peer review) is the author/funder, who has granted bioRxiv a license to display the preprint in perpetuity. It is made available under aCC-BY 4.0 International license.

Figure S3. Extended CAZyme data for Hadza and reference isolates. (A) Number of all GHs and PLs per genome. Color gradient indicates the raw numbers of individual CAZymes of each family present in each genome. Hadza isolates indicated in black, reference strains indicated in gray. **(B)** Number of GHs from all putative mucin-degrading GH families.
